# Healing Childhood Psychological Trauma and Improving Body Image Through Cosmetic Surgery

**DOI:** 10.3389/fpsyt.2019.00540

**Published:** 2019-08-08

**Authors:** Ka Tung Vivianne Ip, Wing Yee Ho

**Affiliations:** ^1^Department of Special Education and Counselling, The Education University of Hong Kong, Hong Kong, Hong Kong; ^2^School of Education and Languages, The Open University of Hong Kong, Kowloon, Hong Kong

**Keywords:** childhood psychological trauma, body image, cosmetic surgery, body satisfaction, addiction

## Abstract

Cosmetic surgery is an interdisciplinary field involving cosmetics and medicine that stems from the early modern obsession with disfigurement. The “correcting” of facial features and body parts was very likely because beauty was at the heart of most reconstructive desires. Cosmetic surgery patients typically experience improvements in body image, and some are very satisfied with the impact of cosmetic surgery in changing their behaviors and improving self-esteem. The doctrine of mind–body connection supports the concept of healing the heart through the body. However, some people feel disgraced after revealing their experiences of cosmetic surgery. It is known that people who experience childhood psychological trauma, such as abuse and school bullying, may opt for cosmetic surgery later in life. The present study aimed to explore the relationship between childhood psychological trauma, cosmetic surgery, and body image. Three female adults who had undergone different types of cosmetic surgery completed the Acceptance of Cosmetic Surgery Scale (ACSS), the Fear of Negative Appearance Evaluation Scale (FNAES), and the Multidimensional Body-Self Relations Questionnaire (MBSRQ), followed by semistructured face-to-face interviews. According to the results, 1) undergoing cosmetic surgery can enhance self-confidence, reduce body dissatisfaction, resolve inner conflicts, and somewhat relieve psychological distress; 2) self-esteem and body image obtained from cosmetic surgery can resolve the distressing aftereffects of childhood trauma that occur later in life; and 3) the perceived sense of beauty achieved from cosmetic surgery contributes to a certain degree of self-confidence in the short term and promotes appearance-enhancing behaviors while increasing the distress of others discovering their cosmetic surgery experiences. The implications of this study are that undergoing cosmetic surgery can have healing effects on childhood trauma; however, there are certain drawbacks that can occur, such as distress and an insatiable desire for or an “addiction” to surgery.

## Introduction

Although it is difficult to define plastic surgery, it can be described as a procedure for the reparation of injury, surgical refinement of deformity, restoration of function, or reconstruction or alteration of the human body (1). Plastic surgery has played a significant role in the healing of wounds and processes of regeneration (2). A study conducted after World War II (3) showed that former soldiers whose faces had been disfigured during war found it harder to find employment, compared to those with intact faces. Thus, plastic surgery is generally chosen by people with burns or injuries (4) in order to reconstruct or camouflage scars, or by those afflicted with congenital disease (5).

Cosmetic surgery can be described as one of the various fields of plastic surgery that mainly focuses on enhancing a patient’s appearance. A previous study (6) reported that the first cosmetic surgery was performed in 16th-century Britain. Later, the innovation of cosmetic facial surgery first arrived between 1970 and 1979 (7). Since then, cosmetic surgery has become a common as well as popular phenomenon, as more people are choosing to undergo a quick transformation in order to fulfill their standards of beauty.

In 2017, a total of 4.31 million cosmetic and nonsurgical procedures were performed in the United States, which was the highest in the world and represented 18.4% of the world’s total. Brazil took second place, with 2.42 million procedures performed in 2017, representing 10.4% of the world’s total. Meanwhile, Japan has also faced criticism for the prevalence of cosmetic practices, with a total of 1.67 million procedures performed, representing 7.2% of the world’s total and ranking third (8). In 2010, the number of cosmetic surgeries performed globally was 14 million (9); however, in 2015, the number has exceeded 21 million, with an increase of over 30% (10). Thus, it is clear that the number of cosmetic surgeries has continued to rise worldwide in recent years.

Influenced by trends in South Korea and Japan, a cosmetic surgery craze has swept across East and Southeast Asia (e.g., China, Hong Kong, Thailand) in recent years, promoted by both mass media (e.g., TV shows, international beauty pageants) and social media (e.g., Facebook, Instagram, and blogs). People choose to undergo cosmetic surgery with the intention of improving certain parts of their body that they feel unsatisfied with. The pursuit of perfection and expectation of resolving intra- and interpersonal conflicts can be the factors leading to cosmetic “addiction.” In 2017, there were 20 million cosmetic procedures performed on women, and 3 million such surgeries performed on men (8). Currently, the majority of patients who undergo cosmetic surgery are women. With the increase in social networking and cosmetic techniques, some specialists [e.g., Ref. (11)] have predicted that the popularity of cosmetic surgery will also gradually increase among the male population.

Plastic surgery helps to heal not only physical imperfections but also psychological wounds. Gaspare Tagliacozzi (12), the father of modern plastic surgery, stated that his practice aimed to “bring back, refashion and restore to wholeness the features which nature gave but chance destroyed, not that they may charm the eye but that they may be an advantage to the living soul’’ (p. 341) (13). Pert (14), a neuroscientist and pharmacologist, stated that “the body and mind are not separate, and we cannot treat one without the other” (p. 274). By undergoing plastic surgery, people aim to simultaneously heal their bodies and minds. One study found that people who underwent cosmetic surgery reported an increase in their self-confidence levels (15) and improvements in their interpersonal relationships (1).

Body image refers to ‘’a phenomenon of a lifetime of ongoing subjective experiences, including memories and emotions that combine with tangible sensory data that are unique to the individual within a broader social and cultural context’’ (p. 83) (16). The collective cognitive and emotional experiences of individuals formulate their body image, which constructs the awareness of the relation of the body with the physical environment, and enables changes in response to new sensory inputs (16), and even constructs new schemas. Body image is influenced by the beliefs, expectations, and prejudices of the individual, as along with the societal and cultural standards of desirable features (17). Individuals combine and internalize the information received from family, peers, and social media to create an ideal body standard for themselves. This standard of measurement evokes self-judgment on his or her image and is manifestly linked to an individual’s emotional state regarding body image.

Prior research has revealed that family (18) and peers (19) are the key figures that affect individuals’ body image, as well as possible contributors to the development of body dissatisfaction (20). Direct parental comments about their child’s body size or appearance formulate their concepts of body image. Peer influence on body image begins in childhood and continues into adolescence and young adulthood. Peer evaluations and teasing may subsequently influence one’s own judgment of beauty and an ideal self-image. Body dissatisfaction during childhood and adolescence can present risks in the development of body image (21), as negative judgment leads to poor body image and body dissatisfaction. Certain studies [e.g., Refs. (22, 23)] have indicated a significant positive correlation between the overall body image, appearance evaluation, and appearance orientation.

Maltreatment by parents (24) and peers (25) has a negative impact on the psychological development of children. Childhood abuse and neglect tend to increase the risk of body dysmorphic disorder in young adulthood (26). People who were abused (27) or emotionally neglected during childhood are more likely to undergo cosmetic surgery later in young adulthood.

Apart from child abuse, school bullying is also one of the most common causes of childhood psychological trauma. Bullying is defined as intentional, repetitive harming or injury by one’s peers (28). The types of bullying include physical (e.g., hitting, pushing, and kicking), verbal (e.g., name-calling and teasing), relational (e.g., social exclusion and spreading rumors), and cyber (e.g., e-mail, instant messaging on personal computers, or text messaging on cell phones) (29). One prior study reported that bullying may lead to victims opting for cosmetic surgery later in life (30).

People who seek cosmetic surgery may be experiencing psychosocial distress (31). Some patients with serious psychiatric disorders, such as body dysmorphic disorder (BDD) and eating disorders, may complain about their smallest appearance flaws or undergo excessive emotional distress due to body image dissatisfaction (31). Some patients seek and become addicted to cosmetic surgery and demonstrate a pattern of repeatedly seeking surgery in order to relieve distress. Cosmetic surgery addiction is a kind of polysurgical addiction (32), in which people demonstrate a compulsive need for surgical interventions. People with such disorders tend to opt for surgical rather than psychiatric treatment (31). Research indicates that, in most cases, cosmetic surgery does not improve the symptoms of BDD (33). Cosmetic surgery has been disparaged as an irrational choice for people who are excessively preoccupied with their appearance.

**Figure 1:**
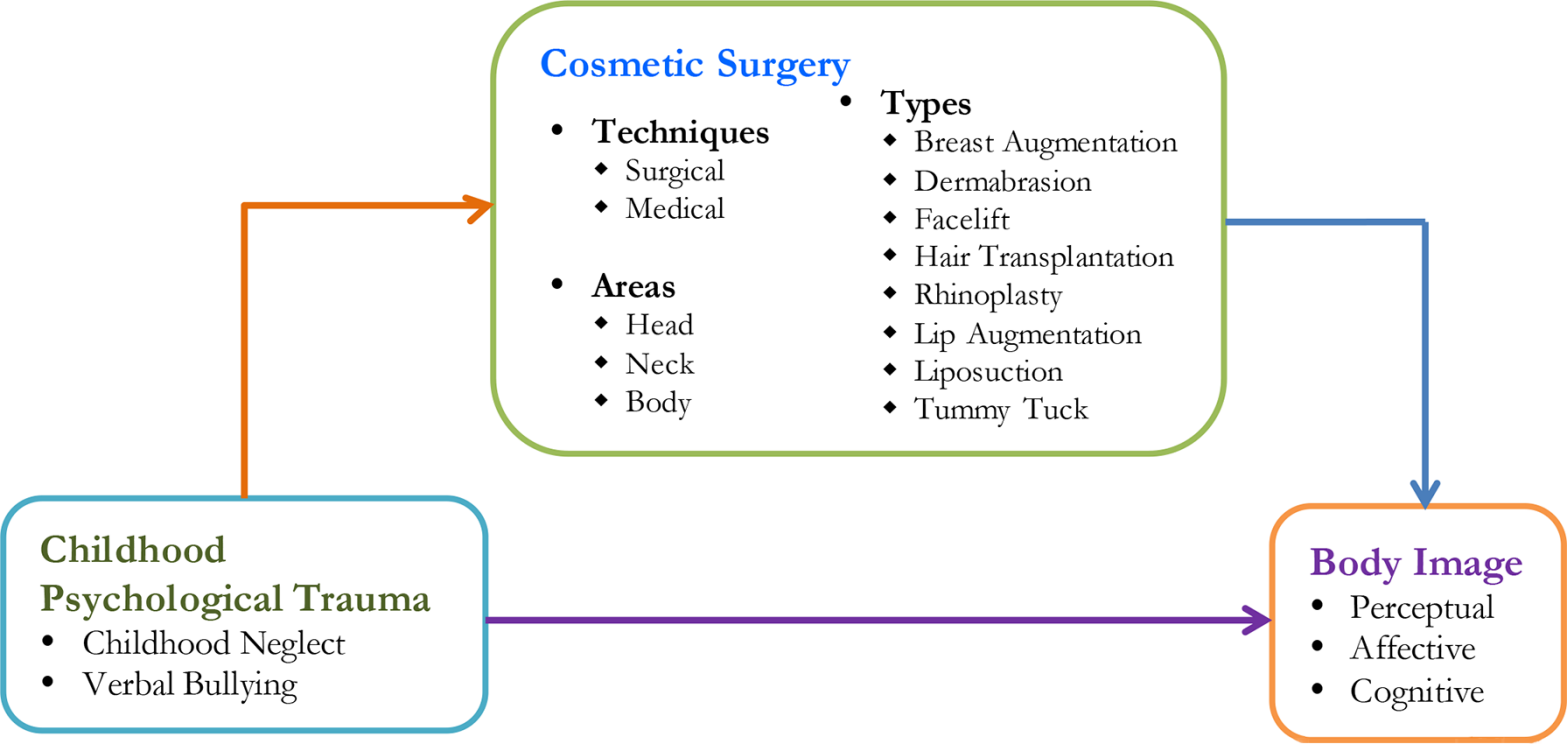
The conceptual framework.

Nevertheless, previous studies [e.g., Ref. (34)] have reported that cosmetic surgery can enhance the level of self-confidence by improving a person’s physical appearance and attractiveness. Cosmetic surgery appears to provide several benefits to people who suffer from body dissatisfaction. Although there are numerous studies [e.g., Ref. (35)] on body image dissatisfaction and BDD, researchers have seldom focused on healing childhood psychological trauma by undergoing cosmetic procedures. A previous study revealed that childhood bullying victims are at an increased risk of undergoing cosmetic surgery in adulthood (30). The findings reported victims experiencing an extreme desire to undergo cosmetic surgery and, consequently, demonstrated significant improvements in depression and anxiety. Another study (36) also stated that cosmetic surgery could have a positive influence on mental health. With the aim to shed light on the controversies surrounding cosmetic surgery, the present study explores the impact of cosmetic surgery on remedying childhood psychological trauma. The conceptual framework shown in **Figure 1** illustrates the various relationships.

## Methods

A mixed-methods case study was designed to assess body image as well as the acceptance of cosmetic surgery while exploring the experience of childhood psychological trauma and cosmetic surgery.

### Participants

Participants were chosen purposively; all had undergone cosmetic surgery. Three female adults, ranging from 25 to 30 years of age (M = 27.67, SD = 2.52), were recruited using the snowball sampling technique.

### Materials

First, interviewees were asked to provide informed consent. Thereafter, they completed the Multidimensional Body-Self Relations Questionnaire Appearance Scales (MBSRQ-AS) (37), Fear of Negative Appearance Evaluation Scale (FNAES) (38), and Acceptance of Cosmetic Surgery Scale (ACSS) (39), in order to measure their attitudinal dispositions toward the physical self, the fear of negative feedback on their appearance, and the acceptance of cosmetic surgery, respectively.

The MBSRQ-AS is a self-report inventory for the assessment of body image. It is a 34-item self-report inventory that consists of five subscales: appearance evaluation (AE) (7 items), appearance orientation (AO) (12 items), overweight preoccupation (OWP) (4 items), body areas satisfaction scale (BASS) (9 items), and self-classified weight (SCW) (2 items); these are measured on 5-point scales (complete satisfaction to complete dissatisfaction). The MBSRQ-AS is used to measure one’s attitudinal dispositions toward the physical self. The three dispositions are evaluative, cognitive, and behavioral components. Moreover, the physical self-encompasses not only measure on one’s physical appearance but also the body’s competence and its biological integrity (40). The internal consistency of the five subscales ranged from 0.73 to 0.89 and test–retest reliability ranged from 0.74 to 0.91 (37).

The FNAES (41) was adopted to measure the fear of negative feedback regarding an individual’s appearance. It consists of a six-item self-report measure that assesses apprehension about appearance evaluation, measured on a 5-point Likert scale (1 = not at all, 5 = extremely). The novel item is index apprehension, which relates to a negative experience with appearance evaluative. It is reliable, with high internal consistency (0.94).

The ACSS (39) involves the multidimensional measure of various aspects of attitudes toward cosmetic surgery. It consists of three subscales. The intrapersonal subscale measures the attitudes related to self-oriented benefits of cosmetic surgery. The social subscale evaluates the social motivations for cosmetic surgery. The consider subscale measures the probability of a participant to consider undergoing cosmetic surgery. All three identified elements are associated with cosmetic surgery attitudes, the degree to which an individual would consider having cosmetic surgery, and the acceptance of cosmetic surgery based on social and intrapersonal motivation. The ACSS is comprised of 15 items for indicating the participants’ level of agreement on a 5-point Likert scale (1 = strongly disagree, 5 = strongly agree). A high score indicates high acceptance of cosmetic surgery; scoring is reversed in the questionnaire. Previous research has shown that the ACSS has high internal consistency, good test–retest reliability after 3 weeks, and good convergent and discriminant validity (39). Cronbach’s alphas for the three subscales were relatively high (intrapersonal 0.92, social 0.90, and consider 0.90).

Second, face-to-face semistructured interviews were conducted individually to understand the experiences and psychological effects of childhood trauma, attitudes toward cosmetic surgery, and cosmetic surgery’s influence on the self and interpersonal relationships.

### Procedure

The study was divided into two parts. First, the interviewees were told about the aims of the study, its benefits, voluntary participation, anonymity and confidentiality, and the right to withdraw. Second, to protect and maximize the benefits for participants, the interviewees were asked to complete a consent form and the questionnaire through email, before participating in the research. To avoid discomfort, the interviewees were given an interview guideline with structured questions regarding early childhood trauma and the experience of undergoing cosmetic surgery before conducting this study. Third, the three interviewees were then invited for two 45-min semistructured interviews. The details of the study were fully explained without deception. The first session of the interview focused on their childhood trauma, such as child abuse and school bullying, whereas in the second session, interviewees were asked to share their experiences, processes, and consequences of cosmetic surgery. The second interview mainly focused on their purposes for choosing to undergo cosmetic surgery, as well as the connection between trauma experience and cosmetic surgery.

All primary qualitative data, such as interview recordings and questionnaires, were kept strictly confidential. The interviewees’ names, photographs, and audio recordings were not disclosed, and the data were only used for research and publication purposes. In addition, to protect and respect the participants’ feelings, referral to a counsellor was provided in case the participants needed help or support after participating in the study.

### Data Analysis

A mixed design was adopted comprising quantitative and qualitative methods for both data collection and analysis. The resulting data were primarily qualitative but included a small quantitative component for tracking the demographic items as well as the three questionnaires. The quantitative data were analyzed using means and standard deviations, while interpretive thematic analysis was used to analyze the qualitative data.

After all interviews were conducted, the audio recordings were transcribed. To establish meaningful patterns, the data were analyzed using a six-phase coding process as follows: familiarization with data, generating initial codes, searching for themes, reviewing themes, defining and naming themes, and producing the final report (42). The researchers read and reread the transcriptions and noted initial ideas related to trauma experience and cosmetic surgery. Then, the transcripts were coded, and the data were categorized with regard to experiences of early childhood trauma, cosmetic surgery, and body image. The transcripts were read, reviewed, and checked by a rater with a background in academic writing; the rater evaluated the coding and classification scheme and checked the transcriptions for accuracy to avoid bias and misinterpretation (43).

In consideration of interrater reliability, the rater and the researchers first coded the transcripts independently and then discussed the coding to find commonalities and divergences in the themes, thereby uncovering the relationships. Such a triangulation process produces convergence and thus supports validity. The themes in the dataset were used to describe and categorize experiences of early childhood trauma, cosmetic surgery, and body image in preparation for analysis.

### Ethical Considerations

This study was approved by the School of Social Sciences Research Ethics Committee (Psychology), Caritas Institute of Higher Education. The research mainly adopted the Ethical Principles of Psychologists and Code of Conduct of the American Psychological Association (44), especially considering principles such as “beneficence and nonmaleficence,” “fidelity and responsibility,” “integrity,” “justice,” and “respect for people’s rights and dignity,” as along with standards such as “resolving ethical issues,” “competence,” “maintaining confidentiality,” “record keeping,” “research and publication,” “assessment,” and “therapy.”

To ensure privacy and confidentiality, the anonymity of each participant’s identity was guaranteed. A consent statement and a declaration were provided, explaining that the interviewees participated voluntarily and had the right to withdraw at any time and did not have to provide a reason.

### Background Information of Interviewees

Interviewee A, a 30-year-old woman, experienced verbal bullying by her male classmates during primary and secondary schools. She currently lives with her boyfriend and has a harmonious relationship with her family. Her family and boyfriend know that she has undergone cosmetic surgery. Beginning in 2010, she underwent a series of cosmetic surgeries, including double eyelid surgery, dark circles treatments, rhinoplasty (nose job), Botulinum toxin injection for face reshaping, plumping cheeks, and V lifting. She feels that she has improved her physical appearance and become a beautiful lady. Now, she visits beauty salons with her friends and reports feeling much better after each cosmetic procedure. Although she has some fear of cosmetic failure, she chooses to not give up the beauty treatments.

Interviewee B, a 28-year-old woman, said she was neglected by her parents during her childhood. She is the eldest sister of the family. Before the birth of her sisters, she felt that her parents loved her very much. However, her parents neglected her and seldom played with her after the birth of her sisters. She complained that she has been assumed to be taking care of her sisters, and sometimes, she feels it is very difficult to be a sister. Her reason for seeking cosmetic surgery is to make herself more beautiful and to look “better.” She has a stable relationship with her boyfriend. Her family and boyfriend are unaware that she has undergone cosmetic surgery. She has already undergone cosmetic surgery twice. The first surgery involved the excision of lesions in sweat glands in order to reduce body odor. The second time was a Botulinum toxin injection for nasal contouring. She worries about the complications and side effects of cosmetic surgery and, therefore, chooses to undergo microplastic surgery.

Interviewee C, a 25-year-old woman, underwent verbal bullying, such as name-calling, by her classmates during primary school. She wishes to please other people in order to gain their approval, for which she kept trying to lose weight and wore makeup daily until it led to a skin allergy. She has undergone cosmetic surgery twice, including medial canthoplasty and rhinoplasty. She has consumed diet pills, used makeup, and enrolled for a slimming treatment. She lives with her family since birth, and her parents and boyfriend are unaware that she has undergone cosmetic surgery. She tends to blame herself while believing that what she does is worthless and that she will always be unhappy, triggering flashbacks of her traumatic experiences. Her skin allergy magnifies her emotional distress.

**Table 1** reports the interviewees’ detailed demographic information, including age, gender, educational attainment, relationship status, and experiences with cosmetic surgery.

**Table 1:** Detailed sample characteristics of interviewees.

Coding ID No.	Age	Gender	Educational attainment	Relationship status	Underwent cosmetic surgery
Interviewee A	30	F	Secondary school	In a relationship	Yes
Interviewee B	28	F	Bachelor’s	In a relationship	Yes
Interviewee C	25	F	Bachelor’s	Single	Yes

## Results

Two interviewees, A and C, reported that verbal bullying was the main factor that led them to undergo cosmetic surgery. Meanwhile, another interviewee, B, reported experiencing childhood neglect. **Table 2** summarizes the results of the MBSRQ-AS, FNAES, and ACSS.

**Table 2:** Summary of the scores, means, and standard deviations of the Multidimensional Body-Self Relations Questionnaire Appearance Scales (MBSRQ-AS), Acceptance of Cosmetic Surgery Scale (ACSS), and Fear of Negative Appearance Evaluation Scale (FNAES).

	Subscale	Interviewee A	Interviewee B	Interviewee C	M	SD
**MBSRQ-AS**	Appearance evaluation (AE)	14	16	14	14.67	1.15
	Appearance orientation (AO)	51	45	50	48.67	3.21
	Overweight preoccupation (OWP)	9	16	15	13.33	3.79
	Self-classified weight (SCW)	3	6	8	5.67	2.52
	Body areas satisfaction scale (BASS)	24	20	23	22.33	2.08
	**Total**	101	103	110	104.67	4.73
**FNAES**	Apprehension about appearance evaluation	18	19	21	19.33	1.53
**ACSS**	Self-oriented benefits	19	18	17	18	1
	Social motivations	17	17	17	17	0
	Consideration of having cosmetic surgery	17	16	18	17	1
	**Total**	53	51	52	52	1

Summarizing the results of MBSRQ-AS, it is seen that there were individual personal factors behind choosing to undergo cosmetic surgery. The findings indicated positive perceptions and feelings of individuals regarding their bodies.

While reviewing the results of the FNAES, we found that all three interviewees chose “Very much” in response to the statement, “It bothers me if I know someone is judging my physical shape.” Furthermore, three respondents chose the answer “moderately” in response to the statements, “I worry that people will find fault with the way I look,” and “I think that other people’s opinions regarding my appearance are too important for me.” The results indicated that all three interviewees were dissatisfied with their body image (physical appearance), and paid considerable attention to their physical appearances and others’ opinions regarding it.

All interviewees’ scores on the ACSS indicated a positive attitude toward the acceptance of cosmetic surgery. The interviewees chose the option “agree” in response to the statement, “Cosmetic surgery is a good thing because it can help people feel better about themselves,” which demonstrates that the acceptance of cosmetic surgery is high. All three of them chose the response “unsure” for the following statement, “I would seriously consider undergoing cosmetic surgery if I thought it would make my partner find me more attractive.” It showed that they took into consideration their partners’ feelings, as their partners may not be able to accept them having undergone cosmetic surgery. Similarly, regarding considerations such as financial issues or side effects, all three respondents chose the answer “unsure” for the statement, “If I can undergo cosmetic surgery for free, I will consider it,” which demonstrates that they may consider undergoing cosmetic surgery if they have enough financial support.

### Childhood Psychological Trauma and Cosmetic Surgery

In the past, cosmetic surgery was performed with the purpose of restoring a disfigurement caused by an accident, illness, or birth defect, which has led to a serious wound, scar, or deformity. Today, the majority of cosmetic surgeries are performed for aesthetic purposes. Research has highlighted numerous factors that may affect the likelihood of undergoing cosmetic surgery, such as religiousness, low self-esteem (45), age, gender, and vicarious experience of cosmetic surgery through family or friends (46). As mentioned above, previous studies have shown that bullying (30) and child neglect (27) may increase the predisposition toward undergoing cosmetic surgery later in life. In this study, interviewees A and C reported their experiences of verbal bullying during primary and secondary school.

The most common name-calling I often heard from classmates was “pork chop” (meaning fat girl). If a girl wore a dress that didn’t fit properly, or even if a girl had a bad haircut, my classmates would discuss it and make fun of him or her for a long time. The difference between males and females is that girls are more concerned about their physical appearance than boys. (Interviewee A)

I had been a fat girl since primary school. I was the only child in my family, so my parents spoiled me a lot. When I was in primary 3, my classmates began calling me “pork chop.” They would always tease me and ask me “do you feel full?” I didn’t really care about them. (Interviewee C)

“Name-calling” is very common in school settings. However, derogatory nicknames may imply a prejudiced view toward others. Interviewee A shared her feelings about her experience of being name-called:

I felt very upset every time a classmate called me “pork chop.” This was because when they called me by this nickname, I would perceive the meaning of “pork chop” to be fat and ugly because they never teased the girls that they liked or called them “pork chop.” (Interviewee A)

A pejorative nickname can destroy self-image and lead to negative feelings. It is a kind of prejudice-based bullying. Bias bullying or prejudice-based bullying is a motivated bullying behavior involving prejudice toward a person’s actual or perceived identity, such as his/her characteristics or circumstance (47). This unfavorable treatment may possibly be a factor leading to undergoing cosmetic surgery during early adulthood.

Interviewee B was affected by the experience of childhood neglect.

I always believed that I had a good relationship with my parents before the births of my two little sisters. My parents began devoting all their attention to them. Sometimes, I feel that my parents are overprotective of my sisters. As the eldest sister, I was taught to assume responsibility for taking care of my sisters. (Interviewee B)

Interviewee B mentioned that she plays an important role in her family. She always reminds herself that she should never disappoint her parents.

I can’t remember how many times while facing difficulties, I haven’t even wanted to share my concerns with my parents. Maybe I know that I am the eldest sister, and thus, I don’t want to put any pressure on my parents. That’s why I never share myself with my family.… They never ask how I feel either. (Interviewee B)

Interviewee B stated that although she has good relationships with both her parents, she does not feel loved by her family.

I think my parents pay too much attention to my sisters. I remember when my two younger sisters would be in trouble, my parents would scold me most of the time. They consider it to be my responsibility to take care of them. I feel so helpless and frustrated because I need parental love and care as well. (Interviewee B)

Appropriate parental care and effective communication are the bridges that connect family members to each other. Interviewee B insisted, “Although I don’t feel loved by them, I really do love them.” Her feelings appear to be complicated and involve a sense of envy. According to Smith (48), envy involves a person who feels inferior noticing others’ superiority or advantage, leading to some form of negative self-evaluation and self-appraisal.

An early adverse experience (e.g., lack of secure attachment, childhood abuse, emotional neglect) may consequently provoke negative emotions, leading to fear of loss, search for secure relationships, as well as emotional scars. Childhood psychological trauma can have a significant effect on children and young adults, with childhood bullying or neglect having the potential to negatively impact their wellbeing. Indeed, the interviewees’ childhood experiences may have had a direct or indirect influence on their appearance enhancing behaviors.

### Undergoing Cosmetic Surgery Enhances Self-Confidence and Body Image

One of the most obvious benefits of cosmetic surgery is perceived improvement in appearance. People with a pleasing appearance tend to have high self-confidence and a more positive body image. The interviewees in the present study shared their first experiences of cosmetic surgery, their internal struggles, and their reflections on the cosmetic procedures.

Interviewee A decided to undergo cosmetic surgery in order to achieve a physical transformation.

I had the desire of undergoing cosmetic surgery since primary school. I couldn’t tolerate the name ‘’pork chop’’ any more, even though I didn’t care how people saw me.… The first cosmetic surgery I underwent was around 7 or 8 years ago. I can’t quite remember how many times I have undergone cosmetic surgery. Every time, I have expected and looked forward to my new look after cosmetic surgery.… I am not a perfect person, but if I can have the opportunity to become a pretty girl, why not try it. (Interviewee A)

Interviewee A believed that cosmetic surgery could give her a beautiful appearance.

I remember the summer vacation between primary six and secondary one, which was a turning point in my life. I started taking slimming pills and underwent slimming treatment. I would make myself aim to become more perfect. (Interviewee C)

Interviewee C reported finding the experience of changing her physical appearance to be pleasurable. While some people may decide to undergo cosmetic surgery to fulfill their desires to look pretty, others may have different reasons.

I think the main reason to undergo cosmetic procedures is to look more beautiful and have a better appearance. I believe this is a common answer for most girls. Of course, I have another reason—I would like to draw the attention of my family.… My parents probably think that I am mature and they don’t have to worry about me. They never seem to care about my feelings, so I try my best to draw their attention. (Interviewee B)

Interviewee B insisted that family reputation was the core factor that motivated her to undergo cosmetic surgery.

The desire, positive feelings, and successful experiences gained from changing one’s physical appearance can be an incentive for reinforcing their appearance enhancing behaviors.

I think that my self-confidence did improve after undergoing cosmetic surgery because I really feel that I am prettier than before. I didn’t feel confident in the past, but now I don’t care how people view me or discuss me. I believe that I will continue to undergo cosmetic procedures in the future. (Interviewee A)

The interviewees believed that cosmetic surgery has a positive impact on enhancing their self-confidence. Same as interviewee A, interviewees B and C also had high self-confidence after undergoing cosmetic surgery. Such pleasurable and successful experiences of cosmetic surgery reduce body dissatisfaction and enhance self-esteem and body image.

### Healing From the Aftereffects of Childhood Psychological Trauma

Cosmetic surgery and other ways changing one’s appearance through the use of cosmetics can enhance self-confidence and body image and may result in healing from some of the aftereffects of trauma that occurred during childhood or young adulthood.

I am not a “pork chop.” No one calls me “pork chop” anymore. I know I look different, but other people don’t know I underwent cosmetic surgery. They admire me and my physical appearance. I am happy to receive their praise. (Interviewee A)

My parents pay more attention to me. They always take me out and introduce me to other relatives. I can see that they are proud of me. They admire how pretty I look. My parents and sisters never ask me about the change in my physical appearance. (Interviewee B)

Interviewees A and B experienced therapeutic effects from cosmetic surgery and improved some of the aftereffects of their childhood trauma. These include no more name-calling or teasing for interviewee A, who is admired by her lover, family, and friends. Likewise, interviewee B gained more attention from her parents. Both of them were able to solve inner conflicts caused by body dissatisfaction.

Interviewee C developed an intimate relationship with a boy from another class after taking diet pills and undergoing slimming treatment.

I was happy to become friends with him and he was the only friend that I knew from secondary school.… Although we were not from the same class, and I knew that he was the only person that I knew from the whole school.… I started thinking about him every day, and I couldn’t believe that I was falling in love with him as I met him again and again! … Unfortunately, he was already in a relationship with another girl. I couldn’t control myself and felt that I was such a bad person. I felt so guilty and sorry for my hidden love! (Interviewee C)

However, when the other classmates started discussing their relationship at school,

He left me alone. I understood that he didn’t want to be involved in this complicated relationship. Although I feel upset, I never blame him. (Interviewee C)

Interviewee C cared about the boy’s feelings to such an extent that she would undergo cosmetic surgery in order to heal the emotional scar and the regret.

I understand that I had made a wrong decision at that moment, I really loved him so much and I really wanted to be with him.… During the graduation dinner, I had full makeup on and was dressed up in a fancy dress. I was looking so gorgeous. By that time, I had undergone cosmetic surgery at least three times.… Cosmetic surgery seems to give me power, a chance, and a new self, which is the most perfect thing in my life. (Interviewee C)

To a certain extent, physical appearance is an important underlying component of self-esteem (49). Cosmetic surgery improves the physical appearance of the body and promotes a positive body image.

### Benefits and Risks of Cosmetic Surgery

People are motivated to undergo more cosmetic procedures because of their internal feelings and desires for beauty (39) and to benefit from secondary gains such as receiving more attention from loved ones or new romantic relationships (50). Although innovative technology can reduce its risks, cosmetic surgery may incur complications, failures, or dissatisfaction. In other words, not all people benefit from cosmetic surgical treatments.

Interviewee C reported more self-confidence after undergoing cosmetic procedures. She believed that she could draw the attention of others, especially her boyfriend. However, she also discovered the dark side of this romantic story.

I remember that the boy asked me out to have sex with him after the graduation dinner, and I understood it was very common to have sex with your partner. However, while we were having sex, he only cared about himself and not my feelings. I realized that he never respected me. He treated me like just a sex partner or only a sex object. (Interviewee C)

People are unaware of how events will unfold until they actually happen. Although interviewee C was relieved of her feelings of regret, she soon came to realize that she had made a mistake. After having experienced the intimate relationship, interviewee C reported that she felt like she had suddenly woken up from a dream. Her love story lasted only a moment, but her sorrow may last a lifetime.

Interviewee C reviewed her decision after this traumatic incident as follows:

In fact, I know it was the consequence and cost of cosmetic surgery. When I reflect back on the years I have spent—the truth, the primary reason for keeping myself fit, for makeup or cosmetic surgery, all these things pop up in my mind immediately. I try to make myself become a perfect girl, I finally realize that nobody seems to notice or appreciate my new body. I was sort of living in a dream until I was hurt by the boy; it was a nightmare and a painful loss. The only thing that came into my mind was.… I never feel happy as I am unlovable and worthless. (Interviewee C)

All interviewees agreed that it is difficult to tell people that they have undergone cosmetic surgery. Therefore, most people choose to not disclose their experiences of cosmetic surgery.

I never tell my parents and my boyfriend that I have undergone cosmetic surgery. If I tell them, I can’t imagine how they would view me … and I don’t know how I would face them as well! (Interviewee B)

I won’t tell anyone that I have the experience of undergoing cosmetic surgery. I prefer to tell people that I am inspired by fashion magazines and makeup skills from Japan. (Interviewee A)

To summarize, childhood neglect and school bullying can harm children and lead to psychological trauma that extends into their future lives. Interviewees A and C stated that they will continue to undergo cosmetic surgery in the future. After undergoing such procedures, both interviewees gained the positive effect of enhanced self-esteem and were able to recover from their childhood psychological trauma.

However, interviewee B experienced complications after cosmetic procedures. She stated that failed cosmetic surgery created a secondary victimization:

I won’t undergo cosmetic surgery again, as it left me with a “shadow” and “scar.” I can use concealer and cover the scar on my face using makeup products, but I will never recover from my psychological trauma. I will not try any other kind of cosmetic surgery again…. I will remember that cosmetic surgery gave me a noticeable scar that can never be repaired. (Interviewee B)

She further stated that she took this experience seriously and would not recommend other girls to undergo cosmetic surgery. Despite her painful experiences, interviewee B agreed that cosmetic surgery does have the positive effects of enhancing self-esteem and body image.

## Discussion

The current study produced three noteworthy findings: 1) undergoing cosmetic surgery had certain positive effects of enhancing self-confidence, reducing body dissatisfaction, resolving inner conflicts, and somewhat relieving psychological distress; 2) self-esteem and body image obtained from cosmetic surgery could help in healing some of the aftereffects of trauma occurring in childhood or young adulthood; and 3) the perceived sense of beauty contributed to improving self-confidence and promoted appearance enhancing behaviors, but increased the distress of others finding out about them having undergone cosmetic procedures.

Cosmetic surgery has been criticized since the end of the 19th century. The earliest cosmetic surgery was performed to reconstruct features of physical appearance, and more recently, it has emerged as a major grooming industry. Its significant role in this age has drawn much attention and concerns regarding the determining of the parameters of physical appearance, especially the appearance of women (51). This study is based on only three case studies, and as such, the limitation of information may be unable to reflect the entire picture.

“The body can and must be healed through the mind, and the mind can and must be healed through the body” (p. 306) (14). People may want to dismiss the significance of appearance dissatisfaction and its subsequent effects on the self, body, and body image. Therefore, this study’s importance lies in making populations more aware of concerns regarding cosmetic surgery.

To conclude, cosmetic surgery has some remedying effects on childhood psychological trauma. Undergoing cosmetic procedures may help restore body image and even heal a broken heart. Although there are several possible psychiatric pitfalls that may arise, cosmetic surgery in some cases is effective in reducing psychological distress resulting from childhood psychological trauma, such as school bullying and childhood neglect. Body image, body satisfaction, and acceptance of cosmetic surgery can be considered potential factors for having a positive impact on people’s minds. The pursuit of beauty is a common trend in society, and the concept of beauty revolves around physical appearance. Thus, cosmetic surgery is no longer considered to be an option only for young women but for men and women of all ages.

The interviewees gained some benefits from cosmetic surgery that promoted their appearance enhancing behaviors. This may pose a risk of cosmetic surgery addiction. In the past, people relied only on cosmetic products to make themselves appear better. However, with the innovation in and continuous development of cosmetic surgery in recent years, people may increasingly decide to seek cosmetic surgery treatments. Thus, people who intend to undergo cosmetic surgery must evaluate their risks, possible aftermaths, and psychological concerns.

Today, cosmetic surgeries can be performed in hospitals, beauty salons, and private clinics. However, to perform such procedures, clinicians do not require a minimum surgical qualification or accreditation. Without regulation or control, not only doctors but also beauty consultants can perform surgery without the necessary knowledge, expertise, and experience. This primarily leaves patients with the responsibility of the quality and consequences of cosmetic surgery. Therefore, rules and regulations for performing cosmetic procedures must be considered.

To some extent, cosmetic surgery does indeed enhance body image and somewhat relieve psychological distress. The data collected here can potentially support further research related to other adverse childhood experiences, such as short stature, amputation, or enucleation. Few prior studies have investigated these topics (52). Future studies could more deeply investigate the influence of cosmetic surgery on adverse childhood experiences as well as its effects in adulthood in terms of body image and psychological adjustment.

## Author’s Note

This study was accepted by the 5th International Academic Conference on Social Sciences (IACSS 2018) and the abstract was presented as a poster format.

## Ethics Statement

This study was carried out in accordance with the recommendations of APA Ethical guidelines, Ethical committee of Psychology, Caritas Institute of Higher Education. The protocol was also approved by the committee.

## Author Contributions

KI and WH designed the study. KI conducted the interviews, transcribed and translated the data, and wrote the original manuscript. WH contributed to manuscript revision. All authors read, revised, and approved the submitted version.

## Conflict of Interest Statement

The authors declare that the research was conducted in the absence of any commercial or financial relationships that could be construed as a potential conflict of interest.

The reviewer LK declared a shared affiliation, with no collaboration, with one of the authors KI to the handling editor.
